# Increasing food insecurity severity is associated with lower diet quality

**DOI:** 10.1017/S1368980024000417

**Published:** 2024-02-05

**Authors:** Katherine Kent, Tracy Schumacher, Sebastian Kocar, Ami Seivwright, Denis Visentin, Clare E Collins, Libby Lester

**Affiliations:** 1 School of Medical, Indigenous and Health Sciences, Faculty of Science, Medicine and Health, University of Wollongong. Wollongong, NSW 2522, Australia; 2 Department of Rural Health, University of Newcastle, Tamworth, NSW 2340, Australia; 3 Institute for Social Change, University of Tasmania, Hobart, Tasmania 7000, Australia; 4 School of Health Sciences, University of Tasmania, Launceston, Tasmania 7250, Australia; 5 University of Newcastle, School of Health Sciences, College of Health, Medicine and Wellbeing, Callaghan, NSW 2308, Australia; 6 Food and Nutrition Research Program, Hunter Medical Research Institute, New Lambton Heights, NSW 2305, Australia

**Keywords:** Food security, Food insecurity, Diet quality, Dietary intake, Australia

## Abstract

**Objective:**

Food insecurity may reduce diet quality, but the relationship between food insecurity severity and diet quality is under-researched. This study aimed to examine the relationship between diet quality and severity of household food insecurity.

**Design:**

A cross-sectional, online survey used the United States Department of Agriculture Household Food Security Six-item Short Form to classify respondents as food secure or marginally, moderately or severely food insecure. The Australian Recommended Food Score (ARFS; scored 0–73) determined diet quality (ARFS total and sub-scale scores). Survey-weighted linear regression (adjusted for age, sex, income, education, location and household composition) was conducted.

**Setting:**

Tasmania, Australia.

**Participants:**

Community-dwelling adults (aged 18 years and over).

**Results:**

The mean ARFS total for the sample (*n* 804, 53 % female, 29 % aged > 65 years) was 32·4 (s d = 9·8). As the severity of household food insecurity increased, ARFS total decreased. Marginally food-insecure respondents reported a mean ARFS score three points lower than food-secure adults (B = –2·7; 95 % CI (–5·11, –0·34); *P* = 0·03) and reduced by six points for moderately (B = –5·6; 95 % CI (–7·26, –3·90); *P* < 0·001) and twelve points for severely food-insecure respondents (B = –11·5; 95 % CI (–13·21, –9·78); *P* < 0·001). Marginally food-insecure respondents had significantly lower vegetable sub-scale scores, moderately food-insecure respondents had significantly lower sub-scale scores for all food groups except dairy and severely food-insecure respondents had significantly lower scores for all sub-scale scores.

**Conclusions:**

Poorer diet quality is evident in marginally, moderately and severely food-insecure adults. Interventions to reduce food insecurity and increase diet quality are required to prevent poorer nutrition-related health outcomes in food-insecure populations in Australia.

## Graphical abstract

**Figure:**
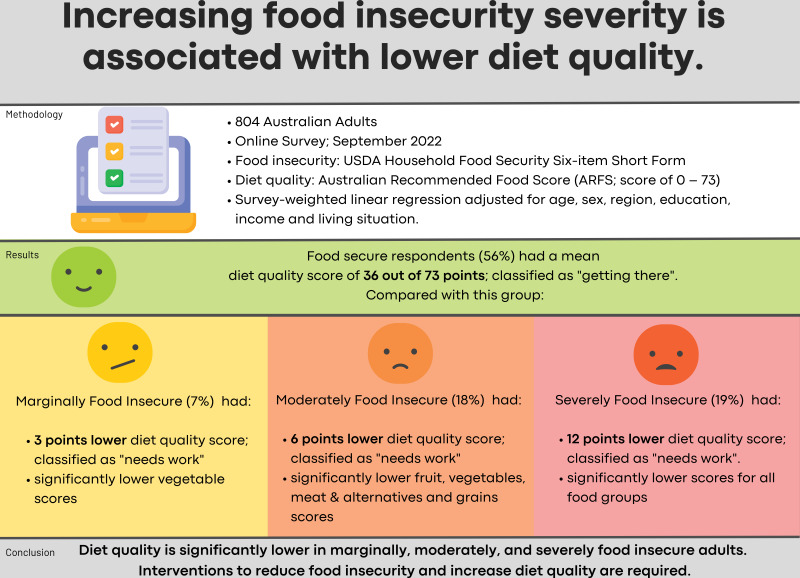

Food security is said to exist when all people, at all times, have physical, social and economic access to sufficient safe and nutritious food that meets their dietary needs and food preferences for an active and healthy life^(1)^. By contrast, food insecurity is the situation when these needs are not met and can range in severity from concern that food will run out, to a reduction in the quality and/or variety of food consumed, to eating less or regularly going without food due to a lack of money for food^(2)^.

A growing body of research in high-income countries indicates that as the severity of household food insecurity increases, the impacts on health and well-being and risk of chronic disease^ ^3^^ are exacerbated^(3)^. Indeed, it has been suggested that people in the USA experiencing marginal food insecurity are more comparable to those with more severe food insecurity, than to those who are food secure^(4)^. This is due to the significantly higher risk for many of the same adverse health outcomes among marginally food-insecure groups^(5)^. Even among population groups already at higher risk of adverse health outcomes, such as low-income adults in the USA, those who were food insecure (to any degree) were more likely than people who were food secure to have clinical evidence of diabetes and hypertension^(6)^. Food-insecure adults, including those in marginally food-insecure households, have reported higher health service use in the USA^(7)^ and Canada^(8)^. The ways in which food insecurity influences health are multifaceted and not particularly well understood^(9)^ and include factors such as stress and other mental health strain^(10)^ and geographic factors such as proximity to grocery stores with a range of affordable fresh food^(10)^. Particularly prominent is the role of dietary factors, where a lack of money for food can constrain food purchasing^(2)^ and alter food consumption patterns^(11)^, leading to compromised dietary intake. There is also emerging evidence that food insecurity in childhood may increase preferences for energy-dense food in adulthood^(12)^.

Food insecurity has been connected with inadequate dietary intake. For example, food insecurity was associated with poorer eating habits and different food procurement and preparation practices when compared with those who were food secure in the USA^(13)^. A 2013 review by Hanson et al. reported that food insecurity was associated with eating less vegetables, fruit and dairy, in addition to a lower intake of a range of micronutrients^(14)^. Since this review, studies have continued to report that food insecurity is associated with poorer dietary intake and diet variety in children^(15)^, young adults^(16)^, low-income adults^(7,17)^ and older adults^(18)^. However, findings about the relationship between food insecurity and various aspects of diet such as nutrient intakes, foods and diet quality is inconsistent^(19)^, indicating that additional examination is required to better elucidate the nature of the relationship between food insecurity and diet quality. Further, a critical limitation of most published studies is that the impact of differing levels of severity of food insecurity on measures of habitual diet quality is less well explored. This is especially evident for marginally food-insecure households, who are sometimes considered ‘food secure’ rather than ‘food insecure’. A graded association between the severity of household food insecurity and measures of diet quality has been reported in Canada^(9)^ and the USA^(19)^. However, this relationship is yet to be explored in other high-income countries, including Australia.

Such an investigation is timely. Household food insecurity appears to be rising across high-income countries including Australia^(20)^, USA^(21)^, UK^(22)^ and Canada^(23)^, related to an increase in climate-related disasters impacting food supplies, supply chain issues following the COVID-19 pandemic and rapidly rising inflation. For example, Kent et al. reported that food insecurity remained significantly higher than pre-pandemic estimates, at 23 % of households in late 2021 compared with 6 % of households in 2019^(20)^. Waxman et al. reported that in June 2022, nearly one-third of American adults faced some degree of household food insecurity within the 30 d prior to the survey, which was comparable to statistics observed during the initial stages of the COVID-19 pandemic. Data released from Statistics Canada’s Canadian Income Survey indicates that there has been an increase in the proportion of individuals residing in households experiencing food insecurity across all provinces in Canada in 2022, surpassing the pre-pandemic estimations from 2019. As the income supports and wage subsidies implemented to deal with the COVID-19 pandemic continue to be dismantled in 2023, and inflation disproportionately impacts the cost of healthy foods^(24)^, it is important to understand and continue advocacy efforts to mitigate the potential negative health and dietary impacts of growing food insecurity. To extend the potential policy impact of food insecurity statistics, further data on the potential dietary deficits associated with food insecurity could be used to advocate and guide policy and practice responses to mitigate the negative effects of food insecurity. Therefore, the aim of this study was to examine the relationship between the severity of household food insecurity and a validated measure of diet quality in Australian adults. We hypothesised that increasing severity of food insecurity would be associated with poorer diet quality.

## Methods

### Study design, setting and population sample

The current study was undertaken as part of The Tasmania Project (TTP), a research project designed to understand the priorities, attitudes and experiences of Tasmanian residents through the COVID-19 pandemic and beyond. Since April 2020, fifteen surveys have been conducted, primarily investigating pandemic-related behaviours and attitudes in addition to topics, such as food, housing, work and well-being. Topics and content of TTP surveys are guided by events and issues in the local context. Previous TTP research has found persistently high food insecurity throughout the first 2 years of the pandemic^(20,25)^, prompting the investigation of the impact of cost-of-living pressures at the time of study on respondents’ food security and diet quality. Therefore, the data from the current manuscript are from the ‘Cost of Living’ survey undertaken between 29 September and 9 October 2022. This study was conducted according to the guidelines of the Declaration of Helsinki and approved by the University of Tasmania Human Research Ethics Committee (Project ID 20587). All participants gave written informed consent. The STROBE-nut checklist was used to design and report the findings^(26)^.

**Fig. 1:**
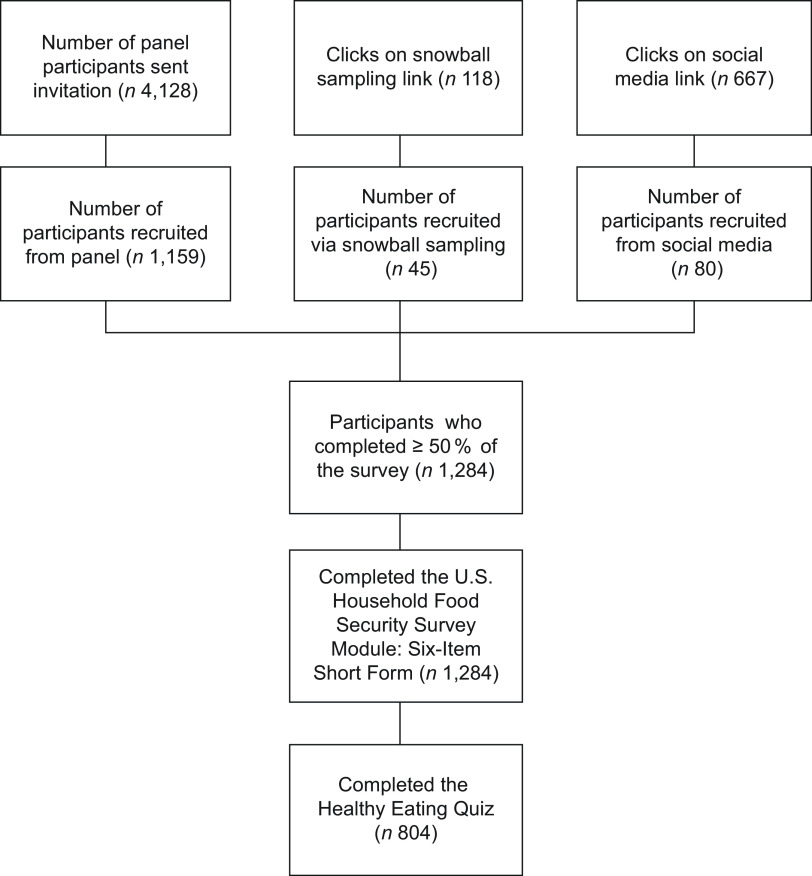
Flow diagram of participant recruitment

Data were collected online via the Qualtrics platform (Qualtrics, Provo, UT). Multiple recruitment strategies were employed (Fig. 1). First, members of the TTP panel were invited via e-mail to complete the survey. The panel comprises people who have provided their e-mail via an online expression of interest form or within prior surveys. A total of 4128 panel members with valid e-mail addresses were invited to complete the ‘Cost of Living’ survey and 1159 (28·1 %) completed at least 50 % of the survey. Social media advertising attracted 667 engagements and eighty survey completions. Lastly, snowball sampling was also used, by inviting respondents to directly share a link to the survey with people they thought may be interested. The snowball link was clicked 118 times, and forty-five people completed at least 50 % of the survey via this link. However, as the number of people who saw the link on social media or the origin of the clicks in snowball sampling (e.g. the ‘recruiter’ could click before forwarding) are unknown, a response rate could not be calculated.

Participants included community-dwelling adult residents of Tasmania, Australia. Participants completed screening questions to confirm residency and that they were aged ≥ 18 years, before being taken to the Participant Information Sheet. Participants provided consent by confirming ‘I have read and understood the Participant Information Sheet and I agree to take part in the project’ before proceeding to the survey. Upon survey closure, data were exported to IBM SPSS Statistics for Windows, version 27·0 (IBM Corp.).

### Household food insecurity status

Food insecurity status was determined using the validated United States Department of Agriculture Six-item Household Food Security Survey Module (HFSSM)^(27)^. The HFSSM includes six questions that explore whether a person has had inadequate food access, availability and utilisation at a household level due to limited financial resources. The six-item screening tool was selected to minimise respondent burden in the survey and for consistency with previous surveys conducted by TTP in this setting^(20,28)^. The six-item screening tool has been validated against the full eighteen-item United States Department of Agriculture survey tool, showing this tool can accurately classify three main categories food security severity (‘food secure,’ ‘food insecure without hunger’ and ‘food insecure with hunger’) in comparison with the longer form^(29)^. The primary limitation of the six-item measure is that it may not capture marginal food insecurity (due to a lack of questions on anxiety over food sufficiency), nor the most severe range of food insecurity including instances of children’s hunger and more severe adult hunger causing weight loss^(29)^. Our study utilised a recall period of the previous 30 d (see^(27)^). Food insecurity status was categorised as a four-level variable, based on number of affirmative responses to the HFSSM. A coding process was used to define marginal, moderate and severe food insecurity, as outlined in Table 1, adapted from the 2021 PROOF Canada report which uses the eighteen-item United States Department of Agriculture HFSSM^(30)^. Marginally food insecure (score = 1) would indicate some income-related barriers to adequate, secure food access; moderately food insecure (score = 2–4; similar to food insecure without hunger) would indicate a compromise in the quality and/or quantity of food consumed and severely food insecure (score = 5–6; similar to food insecure with hunger) would indicate regularly disrupted eating patterns and reduced food intake resulting in hunger. A binary variable was then generated: food secure (a score of 0) or food insecure (score of 1 or more)^(31)^. A manuscript detailing the prevalence of food insecurity severity and coping strategies employed in the full survey sample has been prepared for publication separately^(32)^.

**Table 1:** Overview of coding of food insecurity status using the six-item HFSSM

Food insecurity status		Description	Coding
Food secure		No report of income-related problems of food access.	0 affirmative items
Food insecure	Marginally food insecure	Some indication of an income-related barrier to adequate, secure food access	1 affirmative item
	Moderately food insecure	Compromise in quality and/or quantity of food consumed due to a lack of money for food.	2–4 affirmative items
	Severely food insecure	Regularly disrupted eating patterns and reduced food intake due to a lack of money for food.	5–6 affirmative items

HFSSM, Household Food Security Survey Module; USDA, United States Department of Agriculture.

Descriptions adapted for the six-item HFSSM from a 2021 PROOF Canada report which uses the eighteen-item USDA HFSSM^(30)^.

### Diet quality

The Australian Recommended Food Score (ARFS) is a validated diet quality index consisting of seventy questions that reflect diet variety within core nutrient-dense foods groups and more optimal nutrient intake profiles^(33)^. The ARFS reflects level of alignment with the Australian Dietary Guidelines^(33)^ and has been shown to be a reliable and valid measure of usual diet quality^(33)^. Higher ARFS scores indicate consumption of a greater variety of nutrient-dense foods. It takes approximately 7 min to complete, representing a brief measure of overall diet quality. Eight sub-scales are included and relate to variety in intake of vegetables (0–21), fruit (0–12), meats (0–7), vegetarian sources of protein (0–6), breads and cereals (0–13), dairy (0–11), spreads/sauces (0–2) and water (0–1). Individual questions are scored according to frequency as per the methodology described in Williams et al.^(34)^. Some of the food items for meat (i.e. beef, lamb) and dairy (i.e. ice-cream, frozen yoghurt) had a limit placed on their score for higher intakes due to higher intakes being associated with potentially higher saturated fat or disease risk. Additional points were awarded for greater consumption of vegetables and healthier choices for bread and milk.

Individuals identifying as vegetarian are scored zero for meat protein subscale (out of a total of seven), and instead questions on vegetarian sources of protein are scored double points for meat alternatives, with an additional point awarded if both tofu and lentils are consumed at least once a week, such that total points equate to the same as the meat subscale. User responses were then converted into a total diet quality score (out of a maximum of 73 points) and then categorised into the following groups: ‘needs work’ (< 33), ‘getting there’ (33–38), ‘excellent’ (39–46), or ’outstanding’ (47+).

### Socio-demographic characteristics

Sociodemographic questions were collected including sex (binary; Male, Female), age in years (discrete; categorised into six groups: 18–24 years; 25–34 years; 35–44 years; 45–54 years; 55–64 years; 65 years or older), highest level of education (categorised into High School education or less, Technical and Further Education (TAFE) certificate or diploma, or University level education), postcode (categorised into Statistical Area Level 4 SA4, the largest sub-state region and levels of remoteness using Modified Monash Model 2019 levels 1–7), Aboriginal and/or Torres Strait Islander status (binary; Yes Aboriginal and/or Torres Strait Islander, No), residency status (categorical; Citizen, Permanent Resident, Temporary Resident), Household composition (categorical, recoded into a binary variable of living/cooking alone comprising ‘Person living alone, Non-related adults sharing house/apartment/flat’, and all other couple and family households to capture people living and cooking alone); whether they had a self-reported disability (categorical; No, Yes a little, Yes a lot) and weekly income in $AUD (categorical).

### Statistical analyses

The final sample size was determined by the feasibility of recruitment. Minimally detectable effect sizes were calculated at *α* = 0·05 (significance) and ß = 0·20 (statistical power = 80 %) levels, considering different sample sizes of food (in)security groups that ranged between *n* 56 and *n* 448. The minimum detectable differences for the total ARFS score (range 0–73) were between 2·3 (or 5·0 %, when comparing food-secure and severely food-insecure groups) and 3·7 (or 10·4 %, when comparing food-secure and marginally food-insecure groups).

To improve the representativeness of the sample by mitigating nonresponse and coverage associated bias, data were adjusted post-survey using a raking or iterative proportional fitting calibration technique with sex, age group, highest qualification/education and region (Statistical Area 4) as the weighting covariates (obtained from the Australian Population Census 2021 for the Tasmanian adult population). These four demographic variables are commonly used in TTP surveys to improve the validity of the samples, and the same weighting scheme was used to increase the accuracy of the target variables in this study; for example, ARFS and food security outcome variables are associated with the level of education, i.e. less educated people are more likely to report higher levels of food insecurity and lower levels of diet quality, but are underrepresented in The Tasmania Project panel of participants. The data for the ‘Cost of Living’ survey were raked in R using *anesrake* package.

The remaining statistical analysis was conducted using IBM SPSS Statistics for Windows, version 27·0 (IBM Corp.). Descriptive statistics were used to report the distribution of sociodemographic characteristics and diet quality according to food insecurity status using survey-weighted data. Proportions for categorical variables and means with standard deviations for continuous variables were calculated. Socio-demographic characteristics were compared by household food insecurity status using *χ* ^2^ tests. Linear regression models were fitted to assess differences in ARFS total and sub-scale scores (continuous dependent variables) according to food insecurity status (four-level categorical variable). Data were analysed using an unadjusted model and a model adjusted for age group, sex, region (SA4), education, income and living situation. These confounders were established prior to statistical analysis and were based on literature related to household food insecurity status and dietary intakes^(9,19,34–36)^. As ARFS scores were examined as dependent variables in multiple linear regression models, we excluded ARFS scores for water and sauces due to the need for more than two individual indicators (and they are based on one and two indicators, respectively). Model assumptions were assessed using Durbin-Watson statistics to check for independence of observations. Test statistic values fell within the range of 1·5–2·5, confirming their independence. Variance inflation factors (VIF) were used to examine multicollinearity, with all variables demonstrating a colinear relationship, with VIF statistic consistently < 5 in all cases. Partial regression plots were used to assess potential heteroscedasticity, showing an absence of any discernible patterns or trends and thus confirming homoscedasticity. Normal P-P plots were used to check for normality of residuals and presence of significant outliers. The points in the normal P-P plot indicated that the residuals followed a normal distribution and did not exhibit extreme values or patterns that suggest the presence of influential outliers. These results underscore that no notable violations of model assumptions were found for any of the models presented.

## Results

### Food insecurity status by socio-demographic characteristics

The survey included a total of *n* 804 survey respondents. The survey-weighted estimates indicate that 45 % of participants were assessed as living in households that experienced food insecurity, comprising 7 % marginally, 18 % moderately and 20 % severely food-insecure households. Survey-weighted participant socio-demographic characteristics according to food insecurity status are described in Table 2. More females reported experiencing food insecurity (51 %) compared to males (37 %). Most adults in younger age categories experienced some degree of food insecurity (92 % 18–24 years, 59 % 24–34 years) which reduced with increasing age (Table 2). A smaller proportion of adults with a university education (38 %) were food insecure compared to those with a High School level education (47 %). The highest proportion of food insecurity was evident in Greater Hobart (47 %) the state capital city and lowest in the South-East region (38 %). Food insecurity was higher in adults living alone (63 %) and non-related adults sharing accommodation (70 %) compared with couple families without dependents (28 %). Food insecurity decreased with increasing income categories (Table 2).

**Table 2:** Socio-demographic characteristics of the sample by food insecurity status, survey-weighted for age, sex, geographic region (SA4) and education (*n* (%))

		Total sample		Food secure		Marginally food insecure		Moderately food insecure		Severely food insecure		Total food insecurity		*P* value (χ^2^)
		*n*	%	*n*	%	*n*	%	*n*	%	*n*	%	*n*	%	
Total *n* 804				448	55·7	56	7·0	143	17·8	157	19·5	356	44·3	
Sex *n* 790	Male	371	47·0	236	63·6	15	4·0	43	11·6	77	20·8	136	36·6	<0·001
	Female	419	53·0	205	48·9	41	9·8	99	23·6	74	17·7	214	51·1	
Age category *n* 803	18–24 years	65	8·1	5	7·7	5	7·7	22	33·8	33	50·8	60	92·3	<0·001
	25–34 years	120	14·9	49	40·8	7	5·8	24	20·0	40	33·3	71	59·2	
	35–44 years	115	14·3	61	53·0	11	9·6	18	15·7	25	21·7	54	47·0	
	45–54 years	138	17·2	67	48·6	13	9·4	30	21·7	28	20·3	71	51·4	
	55–64 years	128	15·9	80	62·5	7	5·5	20	15·6	21	16·4	48	37·5	
	65 years or older	237	29·5	184	77·6	13	5·5	29	12·2	11	4·6	54	22·7	
Education *n* 799	High School Education	359	44·9	191	53·2	26	7·2	74	20·6	68	18·9	168	46·8	0·024
	TAFE or Diploma Education	241	30·2	133	55·2	11	4·6	40	16·6	57	23·7	108	44·8	
	University Education	199	24·9	123	61·8	19	9·5	29	14·6	28	14·1	76	38·2	
SA4 *n* 791	Greater Hobart	347	43·9	183	52·7	26	7·5	58	16·7	80	23·1	164	47·3	0·018
	Launceston and North-East	219	27·7	127	58·0	17	7·8	30	13·7	45	20·5	91	41·7	
	South-East	60	7·6	38	63·3	6	10·0	10	16·7	6	10·0	23	37·7	
	West and North-West	165	20·9	96	58·2	7	4·2	41	24·8	21	12·7	69	41·8	
Rurality *n* 765	Inner regional	492	64·3	264	53·7	30	6·1	94	19·1	104	21·1	228	46·3	0·014
	Outer regional	31	4·1	23	74·2	1	3·2	0	0·0	7	22·6	9	28·1	
	Rural or remote	242	31·6	136	56·2	25	10·3	37	15·3	44	18·2	106	43·8	
Aboriginality *n* 792	Not Aboriginal or Torres Strait Islander	754	95·2	434	57·6	54	7·2	129	17·1	137	18·2	320	42·4	<0·001
	Aboriginal and/or Torres Strait Islander	38	4·8	7	18·4	2	5·3	14	36·8	15	39·5	31	81·6	
Citizenship *n* 799	Australian citizen	744	93·1	423	56·9	51	6·9	127	17·1	143	19·2	321	43·1	<0·001
	Permanent resident	27	3·4	21	77·8	0	0·0	6	22·2	0	0·0	6	22·2	
	Temporary resident	28	3·5	4	14·3	5	17·9	10	35·7	9	32·1	24	85·7	
Household Composition *n* 799	Couple with or without non-dependent child (ren)	346	43·3	248	71·7	17	4·9	55	15·9	26	7·5	97	28·1	<0·001
	Person living alone	180	22·5	67	37·2	10	5·6	37	20·6	66	36·7	113	62·8	
	Couple with dependent child (ren)	136	17·0	80	58·8	21	15·4	23	16·9	12	8·8	56	41·2	
	Single parent with dependent child (ren)	20	2·5	7	35·0	0	0·0	3	15·0	10	50·0	12	63·2	
	Multiple family household	51	6·4	24	47·1	3	5·9	9	17·6	15	29·4	26	52·0	
	Non-related adults sharing	36	4·5	11	30·6	2	5·6	14	38·9	9	25·0	26	70·3	
	Other	30	3·8	10	33·3	3	10·0	3	10·0	14	46·7	20	66·7	
Disability Status *n* 797	No Disability	462	58·0	301	65·2	30	6·5	75	16·2	56	12·1	160	34·7	<0·001
	Yes, Life impacted a lot	77	9·7	25	32·5	7	9·1	14	18·2	31	40·3	52	67·5	
	Yes, Life impacted a little	258	32·4	122	47·3	19	7·4	55	21·3	62	24·0	136	52·7	
Income per week category in AUD *n* 782	Negative income	10	1·3	7	70·0	0	0·0	3	30·0	0	0·0	4	36·4	<0·001
	$0	30	3·8	16	53·3	0	0·0	8	26·7	6	20·0	14	46·7	
	$1–$499	202	25·8	69	34·2	14	6·9	48	23·8	71	35·1	133	65·8	
	$500–$999	193	24·7	109	56·5	14	7·3	29	15·0	41	21·2	84	43·5	
	$1000–$1499	136	17·4	75	55·1	8	5·9	33	24·3	20	14·7	60	44·4	
	$1500–$1999	116	14·8	81	69·8	18	15·5	8	6·9	9	7·8	35	30·2	
	$2000–$2999	70	9·0	59	84·3	3	4·3	8	11·4	0	0·0	11	15·7	
	$3000 or more	25	3·2	23	92·0	0	0·0	2	8·0	0	0·0	2	8·0	

All data presented as *n* (%): total sample column presents column percentages, and all other columns present row percentages. *P* value derived from χ2 tests between the food secure, marginally, moderately and severely food-insecure groups. Total food insecurity combines marginally, moderately and severely food-insecure groups. Data survey weighted for age, sex, region (SA4) and education to reflect the Tasmanian Census population.

### Diet quality scores by food insecurity status

**Fig. 2:**
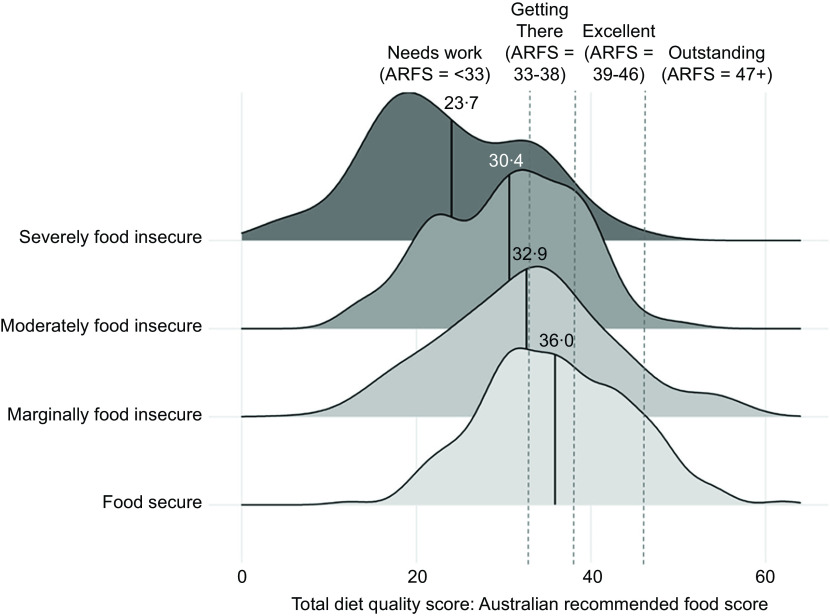
Total diet quality score (Australian Recommended Food Score (ARFS)) by food insecurity status in a ridgeline chart, which presents the distribution of ARFS scores by food insecurity group. The unbroken line is the mean ARFS total score by food insecurity group. Broken lines represent the cut-off points for ARFS scores categorised into four groups of diet quality: ‘needs work’ (< 33), ‘getting there’ (33–38), ‘excellent’ (39–46) or ’outstanding’ (47+)

**Fig. 3:**
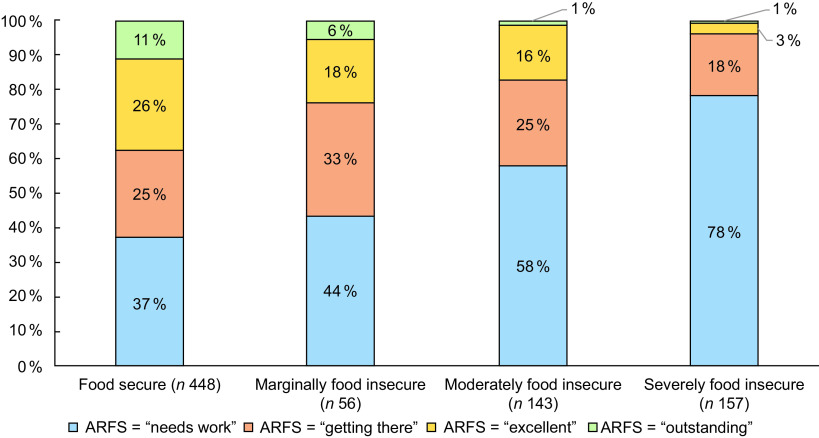
Proportion of respondents Australian Recommended Food Score (ARFS) scores categorised into four groups of diet quality: ‘needs work’ (< 33), ‘getting there’ (33–38), ‘excellent’ (39–46) or ‘outstanding’ (47+) by food insecurity group

The ARFS total survey-weighted mean score for the entire sample was 32·4 (sd = 9·8) (out of seventy-three possible points) (Table 3). ARFS total was 36·0 (sd = 8·4) in adults in food-secure households, which is classified as ‘getting there’ (Fig. 2). ARFS total was lower in households classified as food insecure (mean = 27·8, sd = 9·5). ARFS total score steadily decreased with increasing food insecurity, with participants in marginally food-insecure households (mean = 32·9, sd = 9·5), moderately (mean = 30·4, sd = 8·0) and severely food-insecure (mean = 23·7, sd = 9·1) households all classified as *‘needs work’* the lowest ARFS category (Fig. 2). The food-secure group had the highest proportion of respondents in the ‘*outstanding*’ (11 %) and ‘*getting there*’ (26 %) categories for their ARFS total score (Fig. 3). The proportions of respondents in these categories steadily decreased with increasing food insecurity severity, to 1 % and 3 % in severely food-insecure respondents, respectively. The severely food-insecure group had the highest proportion of respondents in the *‘needs work’* category (78 %), which was also high in moderately food insecure (58 %) and marginally food-insecure groups (44 %).

**Table 3:** Australian recommended food score (ARFS) total and sub-scale scores by food insecurity status using survey-weighted data

ARFS and Sub-scales	Reference range	*n*	Total sample		Food secure		Marginally food insecure		Moderately food insecure		Severely food insecure		Total food insecurity	
			Mean	sd	Mean	sd	Mean	sd	Mean	sd	Mean	sd	Mean	sd
Total Score	0–73	804	32·4	9·8	36·0	8·4	32·9	9·5	30·4	8·0	23·7	9·1	27·8	9·5
Vegetables	0–21	804	12·5	4·5	14·2	3·6	12·0	3·6	11·4	4·3	8·8	4·5	10·3	4·5
Fruit	0–12	803	4·4	2·9	5·2	2·8	5·3	2·9	4·1	2·3	2·5	2·5	3·5	2·7
Meat	0–7	712	3·0	1·5	3·3	1·5	3·1	1·5	2·8	1·3	2·0	1·3	2·5	1·4
Vegetarian sources of protein	0–6	712	2·4	1·4	2·7	1·2	2·6	1·6	2·3	1·4	1·6	1·4	2·0	1·5
Vegetarian proteins (vegetarians only)	0–13	92	8·3	2·9	8·8	2·6	5·8	4·0	7·4	3·1	7·7	3·2	7·5	3·1
Grains	0–13	804	5·3	2·3	5·6	2·3	5·4	2·8	5·3	2·1	4·6	2·1	5·0	2·3
Dairy product	0–11	804	2·9	1·9	3·0	1·9	3·1	2·3	2·8	1·7	2·6	1·7	2·8	1·8
Water	0–1	804	0·6	0·5	0·6	0·5	0·6	0·5	0·6	0·5	0·6	0·5	0·6	0·5
Sauces	0–2	804	0·9	0·8	0·9	0·8	0·9	0·8	0·8	0·8	0·6	0·7	0·8	0·8

Data presented as mean ARFS score (sd), which is a continuous number scored out of 70. Data survey weighted for age, sex, region (SA4) and education to reflect the State Census Population. Further information on scoring of ARFS can be found^(34)^.

Food-secure respondents had higher variety scores for each subscale compared to food-insecure respondents (Table 3). Notably, food-secure respondents scored four points (/21 possible points) higher on average for variety within the vegetable subscale (mean = 14·2, sd = 3·6) compared with food-insecure respondents (mean = 10·3, sd = 4·5). The total sample also had low overall variety sub-scale scores for dairy (mean = 2·9, sd = 1·9/11 possible points). After adjusting for age, sex, education, geographical location (SA4), income and living situation, total ARFS scores were significantly lower for all food-insecure groups compared with the food-secure category, with diet quality scores decreasing as severity of food insecurity increased. The effect size was a three-point lower diet score for marginally food insecure (–2·7 (–5·11 –0·34) ARFS points), six points for moderately insecure (–5·6 (–7·26 –3·90) ARFS points) and twelve points for severely food-insecure respondents (–11·5 (–13·21 –9·78) ARFS points) (Table 4). Marginally food-insecure respondents reported a significantly lower ARFS vegetable score compared with food-secure groups, but there were no significant differences for other sub-scale scores (Table 4). Moderately food-insecure respondents had significantly lower ARFS scores for vegetables, fruit, meat, meat alternatives (for non-vegetarians), grains and water (but not dairy or sauces) compared to the food-secure group (Table 4). Severely food-insecure respondents had significantly lower diet variety scores for all sub-scale scores, except for consumption of meat alternatives for vegetarians (Table 4).

**Table 4:** Regression results for diet quality score by food-insecurity status, unadjusted and adjusted for age, sex, education, geographical location (SA4), income and living situation. Reference category is the food-secure group for all variables

		Unadjusted				Adjusted for age, sex, education, geographical location (SA4), income and living situation.			
Diet quality score	Food insecurity status	B	se	95 % CI	*P* value	B	se	95 % CI	*P* value
Total	Marginally FI	−2·40	1·05	−4·45, –0·35	0·02	−2·72	1·21	−5·11 –0·34	0·03
	Moderately FI	−4·64	0·82	−6·25, –3·03	< 0·001	−5·58	0·86	−7·26 –3·90	<0·001
	Severely FI	−11·41	0·95	−13·2, –9·55	<0·001	−11·50	0·87	−13·21 –9·78	<0·001
Vegetables									
	Marginally FI	−1·14	0·48	−2·08, –0·21	0·02	−1·93	0·54	−2·99 –0·86	<0·001
	Moderately FI	−2·28	0·38	−3·01, –1·54	<0·001	−2·73	0·38	−3·48 –1·98	<0·001
	Severely FI	−5·20	0·43	−6·04, –4·36	<0·001	−4·92	0·39	−5·69 –4·15	<0·001
Fruit									
	Marginally FI	0·02	0·32	−0·60, 0·64	0·95	0·21	0·38	−0·54 0·96	0·58
	Moderately FI	−1·13	0·25	−1·61, –0·64	<0·001	−1·22	0·27	−1·75 –0·69	<0·001
	Severely FI	−2·61	0·28	−3·17, –2·05	<0·001	−2·74	0·28	−3·28 –2·20	<0·001
Meat									
	Marginally FI	−0·24	0·19	−0·61, 0·13	0·20	−0·07	0·20	−0·47 0·33	0·74
	Moderately FI	−0·40	0·15	−0·70, –0·10	0·01	−0·34	0·15	−0·63 –0·05	0·02
	Severely FI	−1·10	0·17	−1·43,–0·76	<0·001	−0·92	0·15	−1·22 –0·62	<0·001
Meat alternatives (not vegetarian)									
	Marginally FI	−0·19	0·18	−0·53, 0·16	0·28	−0·03	0·20	−0·41 0·35	0·87
	Moderately FI	−0·44	0·14	−0·72, –0·16	<0·001	−0·41	0·14	−0·69 –0·13	<0·001
	Severely FI	−1·00	0·16	−1·31, –0·68	<0·001	−1·01	0·15	−1·30 –0·72	<0·001
Meat alternatives (vegetarian)									
	Marginally FI	−1·39	1·29	−3·95, 1·16	0·28	−2·61	2·46	−7·51 2·28	0·29
	Moderately FI	−0·95	0·75	−2·44, 0·55	0·21	−1·12	0·98	−3·06 0·83	0·26
	Severely FI	−1·59	0·94	−3·46, 0·28	0·09	−0·59	0·93	−2·45 1·27	0·53
Grains									
	Marginally FI	−0·47	0·29	−1·04, 0·10	0·11	−0·14	0·32	−0·77 0·48	0·66
	Moderately FI	−0·11	0·23	−0·56, 0·34	0·64	−0·42	0·23	−0·86 0·02	0·06
	Severely FI	−0·70	0·26	−1·21, –0·18	0·01	−0·95	0·23	−1·40 –0·50	<0·001
Dairy product									
	Marginally FI	−0·16	0·23	−0·61, 0·28	0·47	0·00	0·26	−0·51 0·52	1·00
	Moderately FI	−0·38	0·18	−0·73, –0·03	0·03	−0·20	0·19	−0·56 0·17	0·29
	Severely FI	−0·75	0·21	−1·16, –0·35	<0·001	−0·48	0·19	−0·85 –0·11	0·01

## Discussion

To our knowledge, this is the first study to examine a validated measure of diet quality across multiple levels of household food insecurity in Australian adults. The study was conducted during a period of inflation and rising food prices following the COVID-19 pandemic, adding to the novelty and importance of these findings. The results suggest that diet quality decreases as severity of food insecurity increases. People experiencing marginal food insecurity had a lower total ARFS score of approximately three points compared with those who were fully food secure, even after taking income, household composition, geographic region and education levels into account. Total scores were lower by of six and twelve points for those people experiencing moderate and severe food insecurity, respectively. This is an important and large effect size for all food-insecure groups as this much lower score puts their average total diet quality score within the *‘needs work’* diet quality category and represents a usual dietary pattern that is likely to be inadequate in terms of usual nutrient intakes^(33)^. Differences in diet quality for people experiencing marginal food insecurity appear to be driven predominantly by a lower variety of vegetable intakes, whereas variety in intake of fruits, vegetables and meats appears to be lower for people who are moderately food insecure. Variety of intake across all food groups appears to be substantially impacted for those people experiencing severe food insecurity, meaning less intake of nutrient-dense food and therefore worse diet quality.

In our study, nearly one in two households experienced some degree of food insecurity, and concerningly, and one in five households reported experiencing severe food insecurity which is characterised as skipping meals and experiencing hunger. Unlike other high-income countries, Australia lacks routine food insecurity measurement at national level which is one of the factors that hinders the prioritisation and implementation of effective interventions to support food-insecure Australians^(37)^. Current food insecurity estimates at a national level are outdated and/or adopt screening tools that underestimate prevalence^(38)^. More recent estimates of the prevalence of food insecurity have been reported in national surveys conducted by stakeholders from the emergency food relief sector that have adopted non-probability sampling approaches. The prevalence statistics reported by these surveys are comparable to our study, where in 2023, 48 % of households studies reported some degree of food insecurity, up from 45 % in 2022^(39)^. Beyond these national estimates, the prevalence of food insecurity was measured three times by TTP using similar survey methodology to the current study in Tasmania, Australia. It was reported that the age-adjusted prevalence of food insecurity in Tasmania was 28 % in May 2020, 20 % in September 2020 and 23 % in May 2021^(20)^. The increased prevalence of food insecurity reported by this study in late 2022 is likely related to the rapidly rising cost of living caused by inflation in the same time period, during which the price of a healthy basket of food increased by 18 %, including a 13 % increase in the cost of fruits and vegetables specifically^(24)^. Inflation was particularly high in Tasmania, whose capital city Hobart recorded the highest annual inflation among Australian capital cities in the year to 30 September 2022 at 8·6 %^(40)^. In the September quarter alone, food and non-alcoholic beverage inflation was 3·1 % in Hobart^(40)^. Given the large increase in food insecurity found in our study, ongoing, comprehensive food insecurity monitoring by reputable government agencies in representative samples is warranted to adequately inform evidence-based policies and interventions to support food-insecure households in Australia.

In terms of the impact of food insecurity on diet quality, this study reports similar findings to both national and international research. Within Australia, a recent study by Lindberg et al.^(41)^ reported that, on average, food-insecure adults (using a binary food security variable) had a three-point lower total diet quality score (using the dietary guideline index) when compared with food-secure Australians. However, this study was unable to determine food insecurity severity as it utilised a single-item measure, which has also been shown to underestimate food insecurity^(42)^. Another study in Canada reported that as the severity of household food insecurity increased, so did the mean proportion of total energy from ultra-processed foods, which lowered diet quality scores^(9)^. This evidence of decreasing diet quality with increasing severity of food insecurity is also consistent with research in low-income adults in the USA^(19)^.

In the current study, people experiencing marginal food insecurity had significantly lower vegetable variety scores, and lower fruit and vegetable scores were evident for both moderately and severely food-insecure groups. Intake of combined fruits and vegetables has been previously reported to reduce with increasing food insecurity in UK adults^(43)^. A similar association was reported in a representative Korean population^(44)^ and also in children and adolescents^(45)^. These results are supported by literature that suggests the predominant self-reported barrier to fruit and vegetable consumption is the high cost of purchasing these foods^(46)^, and that the availability these foods in the home improves dietary quality^(47)^ and hence lower chronic disease risk.

The lower variety in intake of meat and plant-based proteins reported in our study for moderately and severely food-insecure groups aligns with USA-based research that shows food insecurity is associated with lower diet quality scores for protein foods^(19,48)^. Other research shows mixed findings, which may be related to differences in methodologies for diet quality. For example, a study in pregnant women suggested that increasing food insecurity was associated with greater intake of red and processed meats^(35)^, whereas a study in UK adults suggested only a non-significant trend towards lower protein intake^(17)^ for food-insecure adults. Regardless of food security status, dairy intake by participants in our study was low compared with research in other Australian populations using the same diet quality index (ARFS)^(34,36)^. However, severely food-insecure participants in the current study still had significantly lower dairy variety scores than food-secure participants. This could be explained by the higher cost of purchasing a variety of dairy foods, facilities and costs associated with dairy storage, and that food-insecure households may buy single dairy foods in bulk to save money. Supporting this finding, access to dairy products among Canadian households has shown to be constrained when incomes are low^(49)^, but low-income families tend to spend a higher proportion of their total food budget on milk than other income groups, indicating this food is a priority within their diet^(49)^. Our study also reported significantly poorer diet variety scores for grain-based foods for moderately and severely food-insecure groups. Other research has reported that carbohydrate intakes increase with food insecurity^(41)^, particularly for refined grains^(48)^, which is hypothesised to be related to the relative affordability of these foods compared with animal-based foods, and fruits and vegetables^(50)^. Intake of wholegrains has been shown to be lower among those experiencing food insecurity^(51)^, indicating the importance of examining the quality of carbohydrate-based food consumption rather than absolute macronutrient intake when exploring the relationship between food security, diet and overall health.

These results have implications for the food system in Australia. Australia is more like Canada than the USA in that food relief is generally provided through discrete supports such as food parcels and vouchers in response to situations of financial need, as opposed to broader, federally funded nutrition programs for those who are socio-economically disadvantaged^(52)^. Accordingly, food relief in Australia is primarily focused on alleviating hunger, rather than assuring optimal nutrient intakes through reliable and sustainable access to adequate and nutritious food^(52)^. The current findings that variety in dietary intake differs across food groups and across levels of food insecurity suggest that food relief (and therefore recipients of food relief) could benefit from provision of food with a greater focus on a variety of nutritious foods and particularly greater quantities of items from core food groups. However, as there are long-standing questions about the effectiveness of the food relief system in Australia, and few of our respondents reported accessing emergency food relief^(53)^, the relationship between severity of food insecurity and diet quality reflects a need for further research, evaluation and efforts to develop more sustainable, equitable and nutritious food systems^(54)^. That even the food-secure respondents in the current study were unable to meet dietary quality targets reflecting intakes that align with recommendations in the Australian Dietary Guidelines suggests that there is a substantial role for public health initiatives focused on nutrition education and policy actions to incentivise healthy eating choices (e.g. sugar taxes, junk food advertising restrictions).

Strengths of the current study include the use of a validated measure of diet quality that can capture temporal changes in eating patterns and is preferable compared with studies that utilise single days of dietary records to estimate dietary quality^(34)^ and the use of a multi-item rather than single-item measure of food insecurity. While novel, this study also has several limitations. First, under-reporting is common within self-reported dietary assessment. Also, the ARFS does not measure intake of non-core or discretionary foods, such as ultra-processed foods, limiting comparability with other national and international data. The ARFS is not adjusted for energy, meaning that that individuals with higher ARFS scores may be consuming more food both in terms of quantity and variety. This suggests that a higher ARFS score may not only reflect a healthier food choice but could also indicate a greater overall energy intake. Our use of the six-item HFSSM is unable to determine the full range of experiences of food insecurity including the domains of agency and utilisation due to its focus on financial access to food^(55)^. The short tool may also underestimate marginal food insecurity as it does not ask about anxiety surrounding food procurement like the full eighteen-item HFSSM^(27)^. Additionally, this tool was unable to determine the severity of food insecurity among children. Future research in Australia should consider utilising the full eighteen-item HFSSM which more comprehensively assesses the severity of food insecurity in households for both adults and children^(27)^. As this study recruited participants from a specific geographical area (Tasmania, Australia), the findings may not be generalisable to a broader population. The study employed convenience sampling and snowball sampling, which may have introduced selection bias by relying on easily accessible or self-selected participants. This could compromise the generalisability of study results, as the sample may not accurately represent the larger population, potentially leading to skewed or unrepresentative findings. As survey respondents were generally well educated, older and female, the survey results were weighted to against these factors to compensate for any underrepresented or overrepresented groups. Despite this weighting, non-response bias may not have been fully accounted for meaning our results might not fully represent food insecurity and dietary quality in the Australian population. Finally, as the study was cross-sectional by design, we were unable to determine causality between diet quality and food security status. A call has been made for future aetiologic studies related to food security, diet quality and poor health outcomes to fill this gap^(27)^.

### Conclusion

In conclusion, current findings suggest that food insecurity status is associated with poor diet quality in Australian adults. The dietary quality within several core, nutrient-dense food groups recommended in population level dietary guidelines was sub-optimal in food-insecure adults, including for those experiencing marginal food insecurity. Current results raise questions about how nutritional status of members of food-insecure households will impact future health and chronic disease risk within Australia. The results also underscore the importance of treating marginally food-insecure households as distinct from food secure when examining dietary outcomes. As food insecurity is increasing across high-income countries, urgent efforts are required to improve diet quality in Australian adults experiencing any degree of food insecurity, potentially improving their health outcomes in these groups. Further research examining diet quality and food insecurity in adults and children will be important to extend this research.
